# Volumetric magnetic resonance-guided high-intensity focused ultrasound ablation of uterine fibroids through abdominal scars: the impact of a scar patch on therapeutic efficacy and adverse effects

**DOI:** 10.1186/s40349-017-0100-4

**Published:** 2017-08-17

**Authors:** Bilgin Keserci, Nguyen Minh Duc

**Affiliations:** 1 MR Therapy Division, Philips Healthcare, T Tower, 30, Sowol-ro 2-gil, Jung-gu, 04637 Seoul, South Korea; 2 Department of Radiology, Pham Ngoc Thach University of Medicine, Ho Chi Minh City, Vietnam

**Keywords:** Abdominal scar, Adverse effects, High-intensity focused ultrasound, Magnetic resonance imaging, Scar patch, Therapeutic outcome, Uterine fibroid

## Abstract

**Background:**

To retrospectively compare the treatment success, therapeutic efficacy, and adverse effects of magnetic resonance-guided high-intensity focused ultrasound (MRgHIFU) treatment for uterine fibroid patients with and without abdominal scars.

**Methods:**

Seventy-six women who underwent treatment were divided into group 1 (patients with abdominal scars, which were covered with scar patches that prevents ultrasound energy from reaching the scar tissue immediately behind the patch) and group 2 (patients without abdominal scars). Non-perfused volume (NPV) ratios immediately after treatment, and fibroid volume reduction ratios and symptom severity scores (SSS) at the 6-months follow-up were assessed. All adverse effects were recorded.

**Results:**

The mean NPV ratios in groups 1 and 2 were 87.0 ± 14.1% and 91.5 ± 13.3%. At the 6-months follow-up, the fibroid volume reduction ratios in groups 1 and 2 were 0.45 ± 0.27 and 0.43 ± 0.21, and the corresponding improvement in mean transformed SSS were 0.7 ± 0.39 and 0.79 ± 0.28, respectively. No serious adverse effects were reported. The minor adverse effects encountered in this study are likely related to the temperature increase in the near-field of the ultrasound beam path, which inevitably leads to skin burns, or far-field heat absorption by distant bony structures (i.e., sciatic nerve symptoms), and are typically manifested inter-procedurally and resolved shortly thereafter.

**Conclusions:**

The findings in this study suggest that the scar patch could be used safely and efficiently in MRgHIFU treatment for the patients with uterine fibroids and abdominal scars in the ultrasound beam path.

## Background

Magnetic resonance-guided high-intensity focused ultrasound (MRgHIFU) is a hybrid system combining the therapeutic abilities of HIFU and the imaging capabilities of MR imaging (MRI). It is capable of reducing fibroid size and fibroid-related symptoms, while maintaining an excellent safety profile [1–10]. An immediate non-perfused volume (NPV) ratio of more than 80% in MRgHIFU treatment of uterine fibroids is taken as indicating technical success [9, 10].

Despite the clinical efficacy of this approach for uterine fibroid treatment, the presence of extensive abdominal scars in the ultrasound beam path remains a limitation, due to the potential for skin and subcutaneous tissue burns occurring during HIFU treatment [1].

Uterine fibroid patients with transverse scars could be managed using the following approaches [11–15]: (i) angulation of the transducer to increase the protected area; (ii) using the beam-shaping feature, which reduces the intensity of the ultrasound field in the selected region by shutting off some sonication elements; (iii) using urinary bladder filling to avoid the scar; or (iv) combining these approaches.

However, longitudinal scars are more problematic, as they are usually midline, where the ultrasonic energy has to penetrate through the other intermediate tissue layers. To overcome this problem, acoustic patches on the skin, which can be used to reflect the ultrasound energy from scars, has been introduced [16]. One study [17] reported that the scar patch provides an effective treatment option for patients with abdominal scars located in the beam path, who were previously excluded from MRgHIFU treatment, given the increased risk of skin burns. The safety of a scar patch in MRgHIFU treatment of hypovascular fibroid patients with transverse and longitudinal scars was recently investigated using a volumetric technique [18], and the clinical efficacy was not hampered by the presence of the scar patch.

Therefore, in this retrospective study, we compared treatment success, defined as an immediate non-perfused volume (NPV) ratio of 80%, therapeutic efficacy, defined as fibroid volume reduction, and the symptom severity score (SSS) improvement at the 6-months follow-up, and the safety in term of adverse effects, between patients with and without abdominal scars.

## Methods

### Ethics statement and patient selection

The relevant institutional review board (IRB) approved this study (IRB # 6-CDHA) on May 22, 2015 and written informed consent was obtained from each patient prior to initiation of focused ultrasound-related procedures.

Of the 158 women screened for MRgHIFU treatment, 76 women (age [mean ± SD], 39.2 ± 5.8 years, range, 22–53 years) with 210 uterine fibroids (2.8 ± 3.1 per patient; range, 1–15 fibroids) who underwent HIFU treatment, were divided into 2 groups. Group 1 (n = 21) and group 2 (n = 55) comprised patients with and without scars, respectively. We further subdivided both groups into 2 subgroups, i.e., “subgroup 1”, with an NPV ratio of ≥ 80%, and “subgroup 2” with an NPV ratio < 80%. In group 1, the mean transverse and longitudinal scar lengths were 13.5 ± 5.2 cm (range, 2.6 − 23.2 cm) and 9.1 ± 3.9 cm (range, 3.2 − 14.8 cm), respectively.

Scar tissues in group 1 were covered with polyethylene foam scar patches (QuickCover US Protective Cover, Mectalent Oy, Oulu, Finland; dimensions: 8 mm × 120 mm). These are water-resistant, visible in MRI, suitable for application to scars of different shapes, and remaining immobile during therapy. The patch creates an ultrasound-reflecting air layer, thus preventing ultrasound energy from reaching the scar tissue immediately behind the patch.

Study inclusion criteria were as follows: (1) women aged 18–55 years; (2) clinical diagnosis of symptomatic uterine fibroids; (3) pre- or peri-menopausal status; (4) accessibility of fibroids to MRgHIFU, aimed at complete or near complete ablation (i.e., as close as possible to 100% of the fibroid tumor volume), without sacrificing patients’ safety [19]. Exclusion criteria were: (1) other pelvic diseases; (2) positive pregnancy test results; (3) surgical clips in the direct path of the HIFU beam; (4) contraindication for use of MR contrast agent; and (5) suspected malignancy.

### MRI protocols

All therapies were conducted using a clinical HIFU system (Sonalleve, V2, Philips, Best, The Netherlands) integrated into a 1.5-T MR scanner (Ingenia, Philips).

Images were acquired using (i) a 3D T2-weighted (T2W) turbo spin-echo (TSE) sequence for screening, treatment planning, and 6-months follow-up, (ii) 3D fast-field echo (FFE) for verifying the visibility and the location of the scar and scar patch, (iii) fat-saturated T2W TSE for monitoring abnormally increased signal intensity (SI) in the subcutaneous fat of the abdominal wall, and (iv) a contrast enhanced (CE)-T1-weighted (T1W) sequence for evaluation of fibroid characteristics immediately after MRgHIFU treatment and at the 6-months follow-up. Gd-DO3A-butrol (0.1 mmol/kg; Gadovist, Bayer Schering Pharma, Berlin, Germany) was used for contrast enhancement.

MR thermometry, using volumetric techniques [20], in 3 coronal slices perpendicular to the beam-axis, centered at the focal-region, 1 sagittal slice aligned along the beam direction, and 1 additional slice positioned over the rectus abdominis muscle in the near-field, was achieved using a 2D radiofrequency spoiled gradient-recalled echo-planar imaging (EPI) sequence. Details of MR protocols are presented in Table 1.

**Table 1:** Magnetic resonance imaging sequence parameters

	Screening, planning & follow-up	Scar & scar patch visualization	SI change in the subcutaneous fat	Multiplane MR thermometry	Immediate follow-up^a^
MR Sequence	*T2W 3D TSE with DRIVE*	*T2W 3D FFE*	*Fat-saturated T2W TSE*	*RF-spoiled segmented EPI*	*Fat-saturated T1W THRIVE*
TR (ms)	1300	10	11366	37	5.5
TE (ms)	130	6	70	19.5	2.7
Flip Angle	90	15	130	19	10
Slice Thickness (mm)	1.25	1	4	7	1.5
Matrix	224*218	208*208	200*188	160*100	150*150
FOV ((mm)	250*250	220*220	320*320	400*250	250*250
Number of slices	160	25	36	6	90
Acquisition time (s)	190	47	46	2.9	173
Imaging plane	Sagittal	Coronal	Sagittal	Multi-plane	Coronal
Fat suppression	N/A	N/A	STIR	ProSet	STIR
Additional information	SENSE 2	SENSE 2	SENSE 2	121-binomial water-selective excitation	SENSE 2

*FFE* fast field echo, *T1W* T1-weighted imaging, *T2W* T2-weighted imaging, *SENSE* sensitivity encoding, *EPI* echo planar imaging, *THRIVE* T1W High resolution isotropic volume examination, *TR* repetition time, *TE* echo time, *FOV* field of view, *ProSet* Principle of Selective Excitation Technique, *STIR* short tau inversion recovery, *SI* signal intensity

^a^Gd-DO3A-butrol (0.1 mmol/kg; Gadovist, Bayer Schering Pharma, Germany) was used for contrast enhancement

### MR-guided high-intensity focused ultrasound treatment

The therapeutic ultrasound energy is produced by a 14-cm diameter transducer with a focal length of 140 mm, operating at a frequency of 1.2 MHz. Therapy sonication power levels (70–300 W) were determined by initial test sonication at low power (30–60 W). The treatment cell was ellipsoidal in shape and could be chosen as 4, 8, 12, 14, or 16 mm in axial dimension and 10, 20, 30, 35, or 40 mm in longitudinal dimension. When necessary, urinary bladder filling with normal saline solution and/or rectal filling with ultrasound gel was performed to displace small bowel loops.

Treatment cells were placed on the T2W planning images by carefully considering safety margins from the borders of the treatment cells to the capsule of the fibroid, and to critical organs, such as the small bowel or sacral bone (1 and 4 cm, respectively).

Vital signs, such as blood pressure, heart rate, respiration rate, and oxygen saturation, were also recorded. The patient’s oral or rectal temperature was used as baseline temperature reference during treatment. An oral sedative agent (diazepam 5 mg) was administered 30 min pre-treatment. Intravenous drip infusions of an analgesic agent (paracetamol 1000 mg) and fentanylcitrate 100 μg in normal saline 500 ml were routinely administered before treatment initiation.

### Therapeutic outcome and adverse effects assessment

The immediate NPV ratio was defined as NPV measured in perfusion MR images acquired immediately after MRgHIFU treatment, divided by the fibroid volume measured in T2W images acquired pre-treatment. Any complications or adverse effects, based on self-reporting, were recorded and graded according to the Society of Interventional Radiology (SIR) classification [21], during site visits and telephone contacts.

The percent fibroid volume reduction at 6-months post-treatment was calculated as a proportion of the baseline fibroid volume. The questionnaire included 8 questions of the SSS index, as described by Spies et al. [22]. Transformed SSS at screening and the 6-month follow-up were calculated on a 100-point scale, with higher scores indicating greater symptom severity or bother and lower scores indicate minimal symptom severity.

### Statistical analysis

Values are expressed as the mean ± standard deviation and range for continuous variables and as the number of patients and proportions for nominal variables.

Baseline features, HIFU treatment parameters, rates of complications or adverse effects, and 6-months follow-up results between (i) groups 1 and 2 and (ii) subgroups 1 and 2 in each group, were determined by one-way analysis of variance (ANOVA), or chi-squared and Fisher’s exact tests, as appropriate. P values < 0.05 was considered statistically significant. Statistical analysis was performed using SPSS for Windows (version 24.0, 64-bit edition, IBM, Chicago, IL, USA).

## Results

### Demographic characteristics and MR-guided high-intensity focused ultrasound treatment

Although all fibroids were treated, only the largest fibroid per patient was analyzed. The diameters and volumes of the fibroids in all groups are shown in Table 2. Among the baseline characteristics, the mean subcutaneous fat thickness on the abdominal wall in group 1 was higher than that in group 2 (*P* = 0.002). The anteverted uterus position was predominant in both groups (*P* = 0.003). No other variables differed significantly between the groups. Table 2 summarizes the baseline features of the study population.

**Table 2:** Comparison of baseline characteristics between groups 1 and 2

Characteristics	All patients	Group 1	Group 2	*P* value
Patients	76	21	55	
Ages (years)	39.2 ± 5.8 (22.0–53.0)	40.3 ± 6.0 (29.0–53.0)	38.8 ± 5.9 (22.0–50.0)	0.331
Body mass index (kg/m2)	19.8 ± 1.8 (17.2–25.4)	20.4 ± 2.1 (17.8–25.4)	19.6 ± 1.6 (17.2–24.2)	0.072
Subcutaneous fat thickness (mm)	11.6 ± 4.8 (3.0–26.0)	14.3 ± 5.8 (3.0–26.0)	10.6 ± 3.9 (3.0–20.0)	0.002*
Baseline symptom severity score^a^	52.4 ± 16.1 (21.9–93.8)	51.9 ± 16.2 (21.9–87.5)	52.6 ± 16.3 (21.9–93.8)	0.871
Main Symptoms				
Bulk effect	67	20	47	0.430
AUB	39	10	29	0.799
Uterus position				0.003*
Anteverted	48	19	29	
Retroverted	28	2	26	
Number of fibroid treated (total)	2.8 ± 3.1 (1–15)	2.6 ± 3.0 (1–12)	2.8 ± 3.1 (1–15)	0.805
1fibroid	45	14	31	
2–5 fibroids	18	4	14	
6–9 fibroids	8	2	6	
≥ 10 fibroids	5	1	4	
Diameter (cm)^b^	6.6 ± 2.6 (2.1–15.1)	6.2 ± 2.7 (2.6–15.0)	6.7 ± 2.6 (2.1–15.1)	0.494
Volume (ml)^b^	157.3 ± 141.3 (6.0–794.0)	156.7 ± 164.7 (37.0–7.094)	157.5 ± 133.0 (6.0–637.0)	0.983
Distance (mm)^c^	92.4 ± 17.0 (57.0–133.0)	90.7 ± 13.0 (57.0–117.0)	93.1 ± 18.3 (63.0–1.033)	0.581
Bowel Displacement Technique^d^				0.536
Yes	58	15	43	
No	18	6	12	
Location				0.669
Intramural	38	9	29	
Subserosal	21	6	15	
Submucosal	17	6	11	

Values in parentheses represent ranges

^a^Transformed symptom severity scores (SSS) can range from 0 to 100

^b^Largest treated fibroids only

^c^From Skin to the most posterior part of the largest fibroid

^d^Bowel displacement technique: sequential application of urinary bladder and rectal filling and urinary bladder emptying

*Statistically significant

The mean values of acoustic sonication power in groups 1 and 2, and the corresponding mean treatment durations, measured from the first to the last sonication, are shown in Table 3. The mean treatment speeds were not significantly different between groups (*P* = 0.740). The mean number of therapy sonications used per treatment and the treatment cell size used per treatment did not differ between the groups (*P* > 0.05). Fifteen patients (71.4%) in group 1 and 43 (78.2%) in group 2 required urinary bladder filling (with saline) and/or rectal filling (with ultrasound gel).

**Table 3:** Treatment results of magnetic resonance-guided high-intensity focused ultrasound for groups 1 and 2

Variable	All patients n = 76	Group 1 n = 21	Group 2 n = 55	*P* value
Acoustic sonication power (W)	141.0 ± 25.4 (90–250)	138.6 ± 25.9 (90–180)	142 ± 25.3 (100–250)	0.602
Treatment duration (min)	127.9 ± 55.0 (41–379)	125.4 ± 73.2 (61–379)	128.8 ± 47 (41–258)	0.808
Treatment speed (ml/min)	1.04 ± 0.61 (0.09–2.49)	1.08 ± 0.63 (0.25–2.18)	1.03 ± 0.61 (0.09–2.49)	0.740
Number of therapy sonications per treatment				
overall	33.0 ± 12.2 (14–67)	32.3 ± 15.2 (14–67)	33.2 ± 10.9 (15–65)	0.763
4 mm	9.5 ± 12.1 (1–39)	11.8 ± 18.3 (1–39)	8.0 ± 7.4 (1–17)	0.659
8 mm	13.3 ± 10.3 (1–48)	9.1 ± 8.4 (3–28)	14.6 ± 10.6 (1–48)	0.191
12 mm	11.7 ± 9.3 (1–45)	12.8 ± 8.6 (2–30)	11.2 ± 9.7 (1–45)	0.607
14 mm	17.4 ± 12.7 (1–50)	19.9 ± 14.0 (1–50)	16.4 ± 12.2 (1–39)	0.385
16 mm	16.5 ± 14.1 (1–53)	12.5 ± 14.9 (1–46)	17.7 ± 13.9 (1–53)	0.366
NPV ratio (%)	90.3 ± 13.6 (32.0–100.0)	87.0 ± 14.1 (57.5–100.0)	91.5 ± 13.3 (32.0–100.0)	0.205

Values in parentheses represent ranges

### Immediate treatment outcome

According to immediate follow-up CE-T1W MRI, the mean immediate NPV ratios in groups 1 and 2 were 87.0 ± 14.1% (57.5–100.0%) and 91.5 ± 13.3% (32.0–100.0%; *P* = 0.205, Table 3). In group 1, the NPV ratio was at least 80% in 16 patients (subgroup 1: 93.9 ± 6.6%, 81.1–100.0%), and less than 80% in 5 patients (subgroup 2: 65.2 ± 6.9%. 57.5–74.5%; *P* < 0.001). In group 2, the NPV ratio was at least 80% in 48 patients (subgroup 1; 95.7 ± 6.1%, 80.0–100.0%), and less than 80% in 7 patients (subgroup 2: 62.9 ± 14.5%, 32.0–76.4%; *P* < 0.001).

**Fig. 1:**
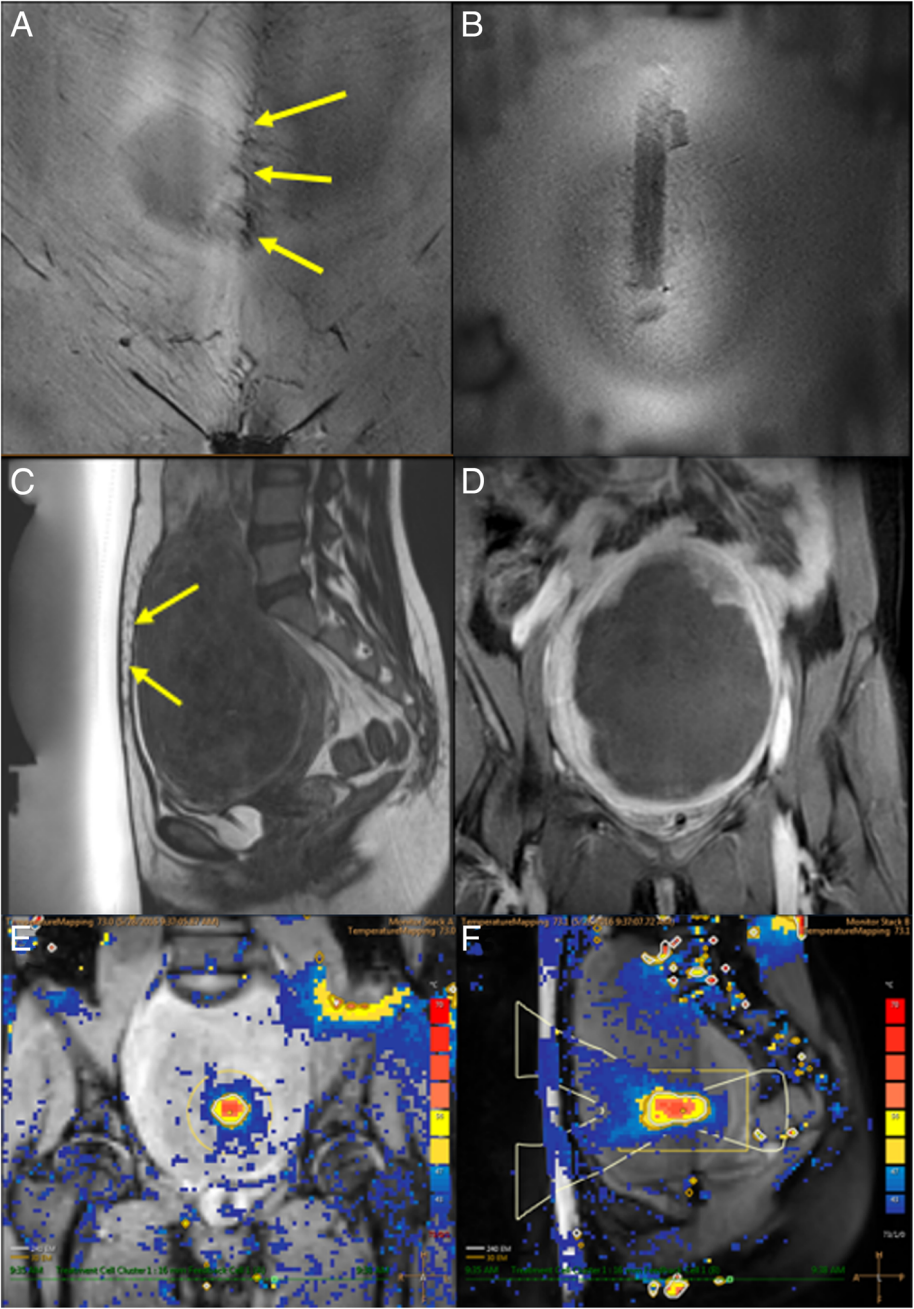
A 35-year-old woman with a 15.0-cm uterine fibroid, who had a 148-mm longitudinal abdominal scar, was treated with MRgHIFU ablation using a scar patch. **a** Scar imaging showing the orientation of the scar within the abdominal fat layer. Scar location identified with yellow arrows. **b** Scar imaging showing the air-containing scar patch at the patient’s skin. **c** Sagittal T2W planning MR image of uterine fibroid prior to high-intensity focused ultrasound treatment. Scar location identified with yellow arrows. **d** CE-T1W image acquired immediately after MRgHIFU treatment. Treatment success in terms of NPV ratio was 92%. **e**, **f** An example of multiplane MR thermometry acquired in both coronal and sagittal planes during one of the sonications

**Fig. 2:**
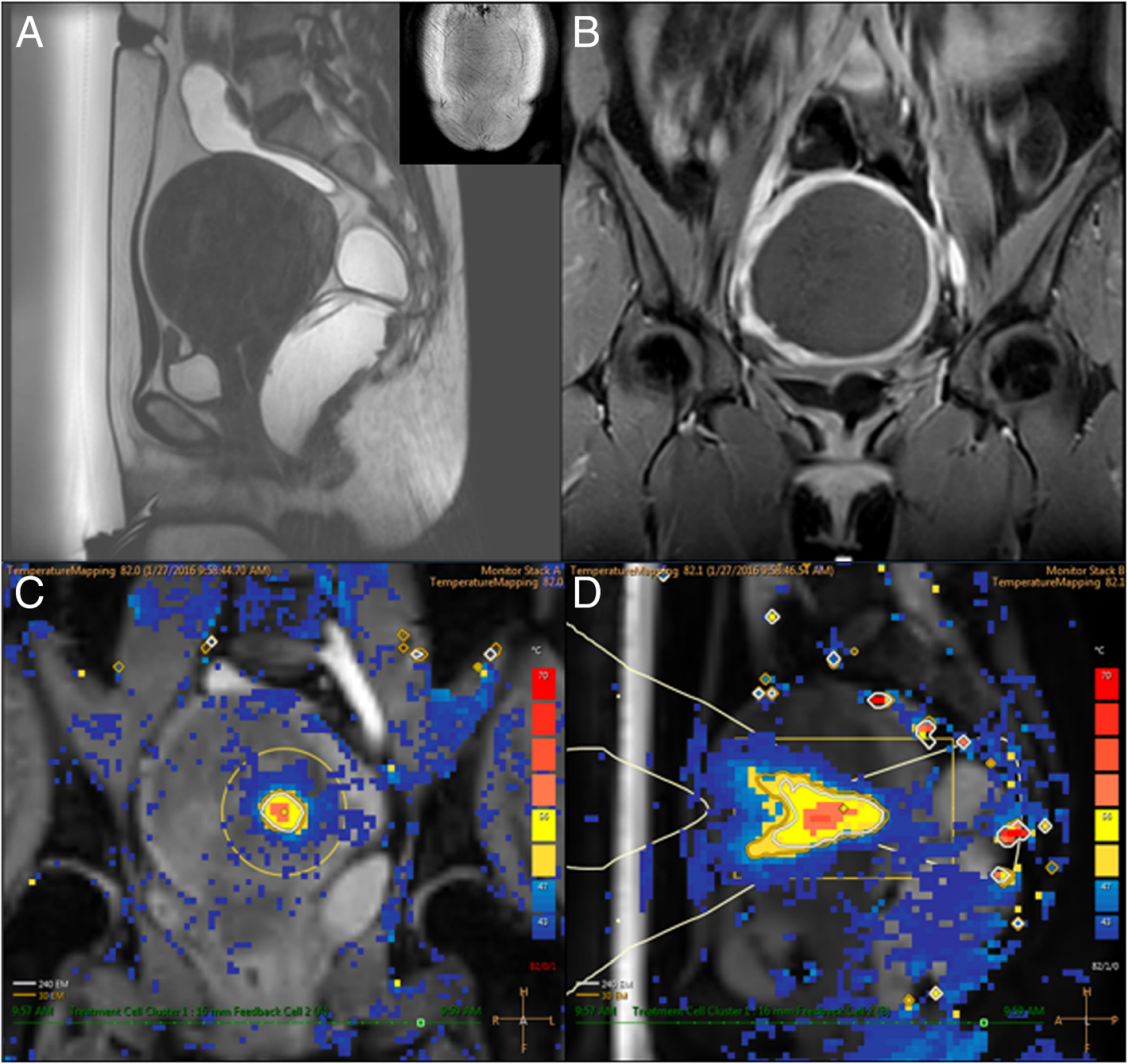
A 33-year-old woman with 10.1-cm uterine fibroid, without an abdominal scar. **a** Sagittal T2W planning MR image of uterine fibroid prior to MRgHIFU treatment. **b** CE-T1W image acquired immediately after MR-guided high-intensity focused ultrasound treatment. The NPV ratio was 100%. **c**, **d** An example of multiplane MR thermometry acquired in both coronal and sagittal planes during sonication

No MRgHIFU therapies were cancelled due to technical failures. Figures 1 and 2 shows examples of the abdominal scar, scar patch, T2W planning, MR temperature mapping, and immediate post HIFU CE-T1W MR images for groups 1 and 2.

### Adverse effects and complications

Fat-saturated T2W TSE MRI showed that 6 patients in group 1 and 15 patients in group 2 had abnormally increased SI in the subcutaneous fat layer, which disappeared within 1–2 weeks without any treatment in 17 patients. However, of the remaining 4 patients, 3 (2 in group 1, 1 in group 2) had a superficial skin burn (first degree) that was resolved within 12 h, without intervention, and 1 in group 2 had a blister (second degree) that was treated conservatively and resolved within 1 week, with 7 days of antibiotics and anti-inflammatory drugs (Amoxicillin − Clavulanic acid, 1 g twice per day, with paracetamol 500 mg twice per day).

Five patients in group 1 and 19 patients in group 2 reported pain during and after MRgHIFU treatment—manifested as mild pain in the pelvic area, back, and buttocks—which was treated by an oral analgesic agent (ibuprofen, 400 mg; thrice per day) and resolved within 3–7 days in all cases. One patient in group 1, and 5 patients in group 2, reported abnormal vaginal discharge and 1 patient in group 1, and 4 patients in group 2, described self-limiting nausea lasting less than 1 h. One patient in group 1 described Foley catheterization-related cystitis symptoms, which was treated with antibiotics (Amoxicillin − Clavulanic acid, 1 g twice per day, for 7 days). Four patients in group 2 described numbness of the leg that had spontaneously resolved after 14–30 days. The heating sensation on the skin with discomfort was observed in 4 patients in group 1, and 9 patients in group 2. The incidence of each type of complication in both groups are shown in Table 4.

**Table 4:** Complications and adverse effects after magnetic resonance-guided high-intensity focused ultrasound ablation for groups 1 and 2

Complications	All patients (n = 76)	Group 1 (n = 21)	Group 2 (n = 55)	*P* value
Minor				
Skin burn grade 1	3 (3.9%)	2 (9.5%)	1 (1.8%)	0.183
Skin burn grade 2	1 (1.3%)	0	1 (1.8%)	1.000
Abnormally increased SI in the subcutaneous fat layer	21 (27.6%)	6 (28.6%)	15 (27.3%)	1.000
Back pain	4 (5.3%)	1 (4.5%)	3 (5.4%)	1.000
Buttock pain	10 (13.1%)	2 (9.5%)	8 (14.5%)	0.717
Cystitis	1 (1.3%)	1 (4.5%)	0	0.276
Nausea	5 (6.6%)	1 (4.5%)	4 (7.3%)	1.000
Numbness foot	4 (5.3%)	0	4 (7.3%)	0.571
Vaginal discharge	6 (7.9%)	1 (4.5%)	5 (9.1%)	1.000
Pelvic pain	6 (7.9%)	2 (9.5%)	4 (7.3%)	0.666
Leg pain	4 (5.3%)	0	4 (7.3%)	0.571
Heating sensation	13 (17.1%)	4 (19%)	9 (16.4%)	0.745
Major	0	0	0	NA

Values in parentheses represent percentages, *SI* Signal intensity

### Therapeutic outcomes at the 6-months follow-up

The 6-months follow-up data were available for only 63 of 76 patients (82.9%) because 13 patients (17.1%; 4 in group 1 and 9 in group 2), were lost to follow-up due to unintended pregnancy (2 in group 1, 1 in group 2) within 6 months of the post-MRgHIFU treatment and the choice to withdraw (2 in group 1, 8 in group 2).

Of the 63 patients, fibroid volume in groups 1 and 2 had decreased from 179.4 ± 176.1 (37.0–794.0) and 168.9 ± 140.2 (6.0–637.0) at baseline to 87.8 ± 68.6 (11.0–295.0) and 94.1 ± 101.6 47.0–578.0) at 6-months post-treatment, corresponding to volume reduction ratios of 0.45 ± 0.27 (−0.03–0.8) and 0.43 ± 0.21 (−0.21–0.84; P = 0.747, Table 5), respectively. The volume reduction ratios at 6-months follow-up in the subgroups are shown in Table 6.

**Table 5:** Comparison of treatment outcome between groups 1 and 2

Treatment outcome	All patients	Group 1	Group 2	P value
Patients	63	17	46	
Fibroid volume^a^				
Baseline	171.7 ± 149.3 (6.0–794.0)	179.4 ± 176.1 (37.0–794.0)	168.9 ± 140.2 (6.0–637.0)	0.806
6 months	92.4 ± 93.4 (4.0–578.0)	87.8 ± 68.6 (11.0–295.0)	94.1 ± 101.6 (4.0–578.0)	0.813
Reduction ratio (6 months)	0.44 ± 0.22 (−0.21–0.84)	0.45 ± 0.27 (−0.03–0.80)	0.43 ± 0.21 (−0.21–0.84)	0.747
Symptom severity score^b^				
Baseline	53.5 ± 16.5 (21.9–93.8)	55.0 ± 15.9 (31.2–87.5)	53.0 ± 16.5 (21.9–93.8)	0.672
6 months	13.0 ± 19.8 (0.0–100.0)	16.5 ± 21.9 (0.0–62.5)	11.7 ± 19.0 (0.0–100.0)	0.391
Improvement ratio (6 months)	0.77 ± 0.31 (−0.2–1.0)	0.7 ± 0.39 (−0.2–1.0)	0.79 ± 0.28 (−0.07–1.0)	0.055

Values in parentheses represent ranges

^a^Largest treated fibroid only

^b^Transformed symptom severe score (SSS) can range from 0 to 100

**Table 6:** Comparison of treatment outcome, based on an immediate NPV ratio of 80%, between groups 1 and 2

Treatment outcome	Group 1			Group 2		
	≥80% (n = 12)	<80% (n = 5)	*P* value	≥80% (n = 40)	<80% (n = 6)	*P* value
Fibroid volume^a^						
Baseline			0.208			0.765
Mean ± SD	214.9 ± 200.0	94.2 ± 33.7		171.3 ± 137.0	152.7 ± 173.6	
Range	37.0–794.0	51.0–143.0		6.0–637.0	12.0–478.0	
6 months			0.925			0.057
Mean ± SD	88.8 ± 79.8	85.2 ± 36.2		83.1 ± 71.0	167.5 ± 215.1	
Range	11.0–295.0	41.0–140.0		4.0–400.0	10.0–578.0	
Reduction ratio (6 months)			0.001*			0.001*
Mean ± SD	0.6 ± 0.15	0.11 ± 0.11		0.49 ± 0.13	0.03 ± 0.16	
Range	0.25–0.8	−0.03–0.2		0.22–0.84	−0.21–0.18	
Symptom severity score^b^						
Baseline			0.916			0.155
Mean ± SD	54.7 ± 14.4	55.6 ± 21.0		51.6 ± 16.1	62.0 ± 17.7	
Range	31.2–75.0	31.2–87.5		21.9–84.4	43.7–93.7	
6 months			0.001*			0.001*
Mean ± SD	4.2 ± 5.0	46.2 ± 16.9		6.2 ± 7.5	47.9 ± 31.8	
Range	0.0–15.6	18.7–62.5		0.0–37.5	6.2–100.0	
Improvement ratio (6 months)			0.001*			0.001*
Mean ± SD	0.93 ± 0.8	0.15 ± 0.27		0.88 ± 0.14	0.25 ± 0.36	
Range	0.75–1.0	−0.2–0.43		0.5–1.0	−0.07–0.89	

*SD* standard deviation

^a^Largest treated fibroid only

^b^Transformed symptom severe score (SSS) can range from 0 to 100

*Statistically significant

The transformed SSS in groups 1 and 2 also decreased from 55.0 ± 15.9 (31.2–87.5) and 53.0 ± 16.5 (21.9–93.8) at baseline to 16.5 ± 21.9 (0.0–62.5) and 11.7 ± 19.0 (0.0–100.0) at 6-months post-treatment, corresponding to improvement ratios of 0.7 ± 0.39 (−0.2–1.0) and 0.79 ± 0.28 (−0.07–1.0; P = 0.055, Table 5), respectively. The transformed SSS improvement at 6-months follow-up in the subgroups are shown in Table 6.

## Discussion

Since scar tissue is less vascular and more fibrotic than normal tissue, the presence of abdominal scars may limit the access to the target area, and also lead to higher temperature increases in the near-field of the ultrasound beam path.

The immediate NPV ratio is one of the most important key parameters for determining treatment success in MRgHIFU treatment of uterine fibroids. In terms of treatment success (as assessed by an NPV ratio of 80%), after delivering similar acoustic sonication power (*P* = 0.602), using a similar number of therapy sonications (*P* = 0.763), treatment duration (*P* = 0.808), and sonication speed (*P* = 0.740), we found no statistically significant difference between patients with and without abdominal scars (*P* = 0.205). Thus, treatment success was not affected by the presence of the scar patch in patients with abdominal scars, concordant with previous studies [3, 4, 6, 8–10, 15, 17, 18].

Among the 63 patients with available 6-months follow-up data, the degree of fibroid volume reduction was not statistically significantly different between patients with and without abdominal scars (Table 5). In subgroup analyses in both groups, fibroid volume reduction in subgroup 1 was significantly greater than that in subgroup 2 in both groups (*P* < 0.001, Table 6), in agreement with one of recent study, which reported 43% and 20% volume reduction at the 3-months follow-up in patients with NPV ratios ≥ 80% and < 80%, respectively [9].

We also demonstrated no significant differences in symptom improvement between the 2 groups (*P* = 0.055, Table 5). In subgroup analyses, patients in subgroups 1 of both groups 1 and 2 exhibited a significantly greater improvement in symptoms than in subgroups 2 (*P* < 0.001, Table 6). While 93.4% of the patients exhibited a decrease in transformed SSS of at least 10 points—considered clinically significant [1, 4, 8, 9]—the remaining 6.6% of patients exhibited a < 10-point improvement in transformed SSS. Our follow-up results demonstrated that therapeutic efficacy was also not affected by the presence of the scar patch in patients with an abdominal scar.

A previous study [17] reported that about 57% of the sonications per treatment passes through the scar patch. In our study, of the 21 patients with abdominal scars in group 1, the percentage of sonications that passed through the scar patch in group 1 were 80.4% ± 18.6 (43.3–100.0%) for transverse scars and 90.6% ± 14.1 (69.6–100.0%) for longitudinal scars (*P* = 0.218). Thus, use of the scar patch might be necessary to treat fibroid patients with both transverse and longitudinal abdominal scars successfully.

To ensure the safety of scar patch usage on these patients, we have further investigated the complications and adverse effects during and after MRgHIFU treatment. The adverse effects encountered in this study are likely related to the temperature increase in the near-field of the ultrasound beam path, which inevitably leads to skin burns, or far-field heat absorption by distant bony structures (i.e., sciatic nerve symptoms), and are typically manifested inter-procedurally and resolved shortly thereafter. As shown in Table 4, most common complaints were (i) back pain due to extended procedure time in the prone position; (ii) leg/buttock pains and numbness due to the location of the fibroid or stimulation of the sciatic nerve by ultrasound sonication energy; (iii) pelvic pain and vaginal discharge due to local edema of the treated region or uterus contraction; and (iv) heating sensation of the skin, abnormally increased SI in the subcutaneous fat layer, and skin burns due to large cell sonications or repetitive sonication of adjacent treatment cells, which caused thermal build-up. Additionally, cystitis was attributed to Foley catheterization.

First and second-degree skin burns were observed in 4 patients. The main reason for skin burn was location of the treatment plane very close to the skin (<60 mm), in order to cover the anterior tumor margin fully in the sagittal plane with the largest treatment cell (i.e., 16 mm in axial dimension and 40 mm in longitudinal dimension), with insufficient cooling time between sonications. These incidents only occurred during the early phase of HIFU treatment, because of lack of operator experience. Later, when the operators had completed their learning curve and gained more experience in patient selection and treatment administration, no skin burns occurred in either group.

These complications are known adverse effects of HIFU of uterine fibroids [1, 6, 9, 10, 23–26] and in line with the results of previous clinical studies [9, 10]. These AEs were regarded as minor complications based on SIR classification. No major complications or severe adverse effects occurred in any of the study patients (https://www.fda.gov/safety/medwatch/howtoreport/ucm053087.htm).

## Conclusions

The present study demonstrated that the scar patch could be used safely and efficiently in MRgHIFU treatment for patients with uterine fibroids and abdominal scars in the ultrasound beam path, who therefore no longer need to be excluded from MRgHIFU treatment. However, to minimize the number of adverse effects, following safety considerations apply: (i) Optimization of the HIFU treatment strategy, e.g., with respect to treatment cell size and location, monitoring temperature increase at the target location and in the near-field of the ultrasound beam path, far-field heat absorption by distant bony structures, sufficient cooling time between each sonication, and selection of acoustic power within a given safety limit. (ii) Fat-saturated T2W TSE MRI scanning for monitoring abnormally increased SI in the subcutaneous fat of the abdominal wall. (iii) Frequent communication with the patient to obtain immediate information about any abnormal sensations.

## Abbreviation

ANOVA: One-way analysis of variance
CE-T1W: Contrast-enhanced T1-weighted
EPI: Echo-planar imaging
FA: Flip angle
FFE: Fast field echo
FOV: Field of view
HIFU: High-intensity focused ultrasound
IRB: Institutional review board
MRI: Magnetic resonance imaging
NPV: Non-perfused volume
NSA: Number of signal averages
ProSet: Principle of selective excitation technique
SD: Standard deviation
SENSE: Sensitivity encoding
SI: Signal intensity
SIR: Society of interventional radiology
SSS: Symptom severity score
STIR: Short tau inversion recovery
T1W-TSE: T1 weighted turbo spin echo
T2W-TSE: T2 weighted turbo spin echo
TE: Echo time
TR: Repetition time
TSE: Turbo spin echo
